# Real‐world completion of diabetes self management education and its impact on patient‐reported outcomes: A scoping review

**DOI:** 10.1111/dme.70312

**Published:** 2026-04-03

**Authors:** Gemma Lewis, David M. Hughes, Greg Irving, John Wilding, Kevin Hardy

**Affiliations:** ^1^ Department of Cardiovascular and Metabolic Medicine, Institute of Life Course and Medical Sciences University of Liverpool Liverpool UK; ^2^ Department of Diabetes and Endocrinology, St Helens Hospital Mersey and West Lancashire Teaching Hospitals NHS Trust St Helens UK; ^3^ Department of Health Data Science, Institute of Population Health University of Liverpool Liverpool UK; ^4^ Health Research Institute Edge Hill University Ormskirk UK

**Keywords:** diabetes, patient education as topic, patient‐reported outcome measures, scoping review, self management

## Abstract

**Aims:**

Diabetes self management education (DSME) is recommended for type 2 diabetes, with completion widely accepted as ≥60% engagement. This review utilising PRISMA extension for scoping reviews maps the literature on the impact of full and partial completion of DSME on patient‐reported outcomes (PROMs) versus a control.

**Methods:**

Core search terms for type 2 diabetes were combined with DSME and PROMs (self care, diabetes distress and QOL) using the AND Boolean operator. Searches of CINAHL, MEDLINE and EMBASE 1 February, 2024, were checked September 2025. No restrictions were applied to study quality, location or sex. We examined quantitative studies comparing outcomes based on DSME completion [100%] vs. control [0%] vs. partial completion [1%–99%]. Studies employing ITT analyses or pre‐post designs without controls were excluded to focus on outcomes by programme completion.

**Results:**

Databases yielded 1307 records with an additional 12 through hand‐searching key journals, reference lists and websites. Twenty‐four records met inclusion criteria. No studies reported outcomes for partial DSME completion. Evidence suggests 100% DSME completion improves self management skills and QOL over 3–6 months, with greater gains in self care behaviours and mental health‐related QOL. Medication adherence and physical health improved less consistently.

**Conclusions:**

Evidence of short‐term improvements in self management skills and QOL was constrained by programme heterogeneity, methodological variability and predominantly high‐income settings. Evidence supporting sustained benefits beyond 6–12 months is limited and impact on diabetes distress is uncertain. No studies reported the effectiveness of partial completion of DSME on PROMs, despite a widely accepted benchmark suggesting ≥60% engagement constitutes completion.

## Graphical abstract

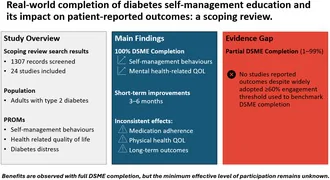

**What's new?**

**What is already known?**

- Diabetes self‐management education (DSME) improves self‐management and quality of life in type 2 diabetes, but evidence is largely based on full programme completion, and recommended completion thresholds (e.g. ≥60%) are not evidence‐based.

**What this study has found?**

- No studies reported outcomes for partial DSME completion. Full completion was associated with short‐term improvements in self‐management and mental health‐related quality of life, with inconsistent long‐term effects and limited evidence for diabetes distress.

**Implications**

- Current completion benchmarks lack empirical support; future studies should evaluate dose–response relationships and report outcomes by level of engagement.

## 1 INTRODUCTION

Diabetes is a complex condition leading to disability and premature mortality with 7000 excess deaths in the United Kingdom in 2022; evidence suggests that many of these deaths were preventable, largely due to delays in routine care.^1^ Globally the number of adults living with diabetes has risen sharply over the past 40 years^2^ with the International Diabetes Federation estimating 537 million people are affected worldwide^3^ of whom approximately 4.3 million reside in the UK.^1^ Over 90% have type 2 diabetes, with an increased prevalence and multimorbidity risk in areas with high deprivation, poor healthcare access, poorer housing, reduced finances and lower educational attainment.^1^

The UK National Diabetes Audit suggests 71% of people with type 2 diabetes are offered diabetes self management education (DSME), with an average attendance rate of 7%^4^ despite National Institute for Health and Care Excellence (NICE)^5^ and the American Diabetes Association (ADA) and European Association for the Study of Diabetes (EASD) stating that DSME is the foundation for successful diabetes management and prevention of diabetes‐related micro‐ and macrovascular complications.^5^, ^6^

Landmark studies have demonstrated the effectiveness of DSME in improving self management skills, reducing diabetes distress and improving health‐related quality of life, along with short to medium term improvements to glycated haemoglobin (HbA1c).^7^, ^8^, ^9^, ^10^ NHS England^11^ and the Centre for Disease Control and Prevention^12^ benchmark education ‘completion’ as ≥60% attendance, yet little is known of the impact of partial completion of DSME programmes on HbA1c or psychometric outcomes. These completion thresholds are defined by implementation guidance and commissioning requirements rather than evidence of an effective educational ‘dose’. One small retrospective study in the USA using secondary data analysis of 105 patient records reported that patients who attended 1 or 8 h or more of DSME had a significant reduction in HbA1c when compared to those who received no education.^13^

Evidence from trials demonstrating DSME benefits is based on full (100%) programme completion.^8^, ^9^, ^14^, ^15^, ^16^, ^17^, ^18^ Similarly, in diabetes prevention programmes, each session attended was associated with a further reduction in body weight of 0.26–0.3%^19^ suggesting greater benefit with higher attendance. The impact of DSME on psychological and emotional health remains unclear, as most studies employ intention‐to‐treat (ITT) analyses which may dilute the effects observed in participants who fully complete the program and do not adequately address outcomes for partial‐completers.

This scoping review aims to map the literature on the impact of full and partial completion of DSME on patient‐reported outcomes (PROMs) compared to controls. Given the observational nature of this research question and the lack of standardised global practices in DSME delivery, a scoping review was identified as the most appropriate method to understand the extent of literature available and identify gaps in knowledge.

## 2 METHODS

This scoping review followed the five‐stage process for scoping studies methodological framework^20^ and was developed using the Preferred Reporting Items for Systematic Reviews and Meta‐Analysis Extension for Scoping Reviews^21^ (PRISMA‐ScR) guidelines. Using a population, intervention, comparator, outcome (PICO) framework as recommended by PRISMA‐ScR, the population comprised adults living with type 2 diabetes; the intervention was structured DSME (full or partial completion); the comparator was no DSME (control); and outcomes were self management skills, health‐related quality of life (HRQOL) or diabetes distress.

### 2.1 Research question

What is the extent of literature that exists about the impact of differing completion rates of DSME on patient‐reported self management skills, health‐related QOL and diabetes distress?

### 2.2 Information sources and searches

To identify potentially relevant evidence, the following bibliographic databases were searched: CINAHL, MEDLINE and EMBASE. Search strategies were drafted and refined with support from an experienced librarian before being conducted on the 1 February 2024 and checked in September 2025. Core search terms for type 2 diabetes were combined using the Boolean operator AND with terms relevant to DSME and PROMs. We identified primary studies and grey literature relevant to DSME for type 2 diabetes. Systematic reviews were initially included and used to confirm that papers potentially meeting the inclusion criteria had not been missed by our search. Final reference lists were exported into Rayyan software^22^ and duplicates removed. Reference lists of final documents were reviewed to identify other forms of literature. Final search strategies for CINAHL PLUS and MEDLINE can be found in supplementary information.

### 2.3 Literature selection

A two‐stage process was adopted for the screening of potentially relevant documents. To ensure consistency throughout the screening process, two reviewers (GL and KH) reviewed the same 20 publications. Agreement between reviewers was assessed using Cohen's kappa (κ = 0.71); discrepancies were discussed and the screening strategy refined prior to full title and abstract screening. Subsequently, the titles and abstracts of all eligible articles were independently and blindly screened by both reviewers using Rayyan software^22^ with inter‐rater agreement improving to *κ* = 0.81. Documents were only excluded at this stage if they did not pertain to DSME. If a title appeared relevant, but no abstract was available, this article was advanced to the second stage. Any conflicts were discussed, amended and where agreement could not be reached a third reviewer was available, although this was not required during this scoping review. During the second stage, two reviewers (GL and KH) independently reviewed all full text articles against the inclusion and exclusion criteria. Authors of six papers were contacted for further information.

### 2.4 Inclusion and exclusion criteria

This review included studies involving adults aged 18 years or older with a confirmed diagnosis of type 2 diabetes.

Pre‐post‐test studies without a control or comparator group were excluded. Although these studies may report changes following DSME, they lack control for confounding factors, limiting causal inference about the effectiveness of DSME relative to programme completion. Excluding these studies helps reduce overestimation of effects, ensuring a focus on comparative evidence and improves the interpretability of findings regarding the differential impact of full versus partial DSME completion.

All quantitative studies which compared outcomes between groups based on DSME attendance or completion levels were considered, including intervention versus control studies and observational studies with comparator groups (e.g. full [100% attendance] vs. partial completion [1–99% attendance] vs. control [0%]) where outcomes for type 2 diabetes were reported independently of other forms. This allowed for exploration of the potential dose–response relationship between DSME attendance levels and outcomes. It is recognised that prevalence, risks and approaches to supported self management differ widely.

Studies that adopted ITT analyses for DSME—those who partially completed or did not attend were analysed together with full completers—were excluded. The focus of this review is on evaluating the effectiveness of DSME based on actual participation (i.e. a per‐protocol analysis). While including ITT analyses are standard and appropriate for drug trials, their use in educational studies like DSME can obscure potential ‘dose’ effects. Specifically, analysing all participants regardless of attendance makes it difficult to assess how differing levels of engagement (e.g. full vs. partial attendance) impact PROMs. Including ITT‐based studies alongside per‐protocol analyses would increase heterogeneity and complicate interpretation. Notably, none of the excluded ITT studies reported a separate analysis for completers as a secondary outcome.

Our review was not specific to gender, sex or ethnicity. Studies published from 2004 onwards were included although we recognise that DSME delivery may have changed over the last 20 years, especially given the introduction of digital programmes. Studies were included where they were written in English, or where an English transcription was available.

DSME is the term recognised for behavioural interventions to support patients' self management skills in type 2 diabetes, however there is no nationally or internationally agreed content. To be classified as DSME for this review the study had to indicate that the programme was evidence‐based, structured for example, approved curriculum with stated learning outcomes and objectives and delivered by trained educators and fundamentally different to patient advice given during a routine consultation. Programmes did not need to be accredited.

Studies were excluded where the focus was on diabetes prevention, carbohydrate counting, type 1 diabetes or where the focus was comparing different methods of delivering education rather than the outcome of DSME education. In cases where study designs included comparisons between control, DSME and DSME combined with medication or additional interventions, only the data comparing control versus DSME were extracted during the review process.

For the purposes of this paper, full completion was defined as participants attending 100% of the sessions offered. Studies could include participants who partially completed the programme, provided that outcomes were analysed on a per‐protocol basis and ITT analyses were not adopted. The review focus was not programme duration, but whether participants who completed all available sessions (‘full completion’) achieved different outcomes compared with those with partial completion when compared to a control. Attendance at a single‐session programme was therefore considered equivalent to 100% completion, consistent with the operational definition applied across study designs. Papers were included where participants had completed DSME but may not have attended all follow‐up data collection appointments, providing an ITT analysis was not adopted.

### 2.5 Data charting process

A data charting table was developed by the first reviewer (GL) prior to being discussed, checked and piloted by two reviewers (GL and KH) on a random sample of 10 articles and modified as required based on feedback. Full data abstraction began only after agreement had been reached. GL conducted data extraction with KH providing verification of included papers, discussing results in an iterative process aligning with the review focus. Studies were grouped by outcome of interest and summarised by setting, study design, outcome measures and author conclusions. Details of study characteristics are summarised in Table 1. For this scoping review only three PROMs were considered: (1) self management skills (encompassing self care), (2) HRQOL (encompassing mental and physical health‐related components) and (3) diabetes distress (excluding generalised anxiety or depression). It is however appreciated that these domains overlap, and diabetes distress can influence overall scores on tools like the Hospital Anxiety and Depression scale. Table 2 presents PROM outcomes.

**TABLE 1:** Study overview.

Author & year	Country	Aims	Sample description at baseline	Outcome measures (power)	DSME characteristics	Author conclusions and recommendations
Bukhsh, 2022^23^	Pakistan	Impact of pharmacist‐led DSME on glycaemic control, self‐care and disease knowledge of patients with poor glycaemic control	Purposive sampling Single site 75 participants (intervention 38; control 37) Mean age 52 (over 30s only) 46% women	HbA1c (80% power to detect 1% change HbA1c at *p* = 0.05) Diabetes knowledge, self management, self‐efficacy	Name of programme not stated Face to face, group format Delivered by pharmacist 2 sessions totalling 1 h Measure of completion not stated, 33/38 intervention participants finished trial	DSME resulted in significant improvements to HbA1c and enhanced disease knowledge, and self care skills
Cai, 2016^39^	China	Effectiveness of a family‐based DSME on HbA1c, BMI, waist circumference, diabetes knowledge, self‐efficacy and self‐care	Convenience sampling Single site 60 participants (intervention 30; control 30) Mean age 65 61% women	HbA1c (80% power to detect a medium effect *f = 0.39* at *p* = 0.05) BMI, waist circumference, plasma lipid profiles, self‐efficacy, diabetes knowledge, family support and quality of life	Family‐based DSME Face to face, group format with two additional 1:1 visits Delivered by healthcare professionals 7 sessions totalling 7 h Measure of DSME completion not stated. 29/30 intervention participants finished trial	Family‐based DSME may help patients with type 2 diabetes modify lifestyle and perform self care activities, improving HbA1c management
Celik, 2022^33^	Turkey	Effectiveness of DSME on self‐care and glycaemic control in type 2 diabetes	Convenience sampling Multi‐site including 28 hospitals 1492 participants (received DSME in last 12 months 1205; no DSME in last 12 months 330) Mean age 56 55% women	Self management skills (95% power to detect 0.4 change at *p* = 0.05) HbA1c	Name of programme not stated Face to face, not stated if group or 1:1 format Delivered by diabetes nurse Between 1 and 3 sessions, length not stated Measure of DSME completion not stated. 1 session deemed enough for some patients, other required up to 3 sessions—patient dependent. 1205 intervention participants finished trial	Education status (received/not received) and frequency are correlated with improved self care Attending at least twice was associated with significantly better self care levels
Changsieng, 2023^25^	Thailand	Impact of DSME on self‐care skills, change behaviour and HbA1c control in Thai adults with uncontrolled diabetes	Purposive sampling Multi‐site including 4 hospitals 120 participants (intervention 60; control 60) Mean age 51 65% women	HbA1c (80% power to detect 0.56 change HbA1c at *p* = 0.05) Self management, dietary consumption, physical activity levels, medication adherence	Programme name not stated (based on Orem's self care deficit nursing theory) Face to face, not stated if group or 1:1 format Delivered by nurses 4 sessions, totalling 6 h Measure of DSME completion not stated. 51/60 intervention participants finished trial	DSME improved knowledge, changed behaviours and reduced HbA1c levels in patients with poorly controlled diabetes
Chen, 2019^32^	China	Impact of DSME on blood glucose and health‐related quality of life	Convenience sampling Multi‐site including 22 health centres 423 participants (intervention 213; control 210) Mean age 63 80% women	Primary outcome measure not stated (not stated) HbA1c, health‐related quality of life	Programme name not stated Face to face, group format Delivered by doctors, nurses and diabetes specialists Number of sessions and total length of programme not stated Measure of DSME completion not stated. 182/213 intervention participants finished trial	DSME improved some elements of quality of life although results differed based on sex, education level and employment type
Davies, 2008^9^	UK	Effectiveness of DSME for newly diagnosed type 2 diabetes	Purposive sampling Multi‐site including 207 general practices 824 participants (intervention 437; control 387) Mean age 60 45% women	HbA1c (90% power to detect 1% change HbA1c at p0.05 12 months later) blood pressure, weight, blood lipid levels, smoking status, physical activity, quality of life, beliefs about illness, depression and emotional impact of diabetes	DESMOND Face to face, group format Delivered by healthcare professionals 1 session totalling 6 h DSME completion measured at 100% attendance. 437 finished intervention. Follow‐up meetings varied in attendance with missing outcomes not replaced, instead a derived average over time of continuous outcomes was calculated	DSME for people newly diagnosed with type 2 diabetes resulted in positive improvements to beliefs about illness, weight loss and smoking cessation, however no difference to HbA1c at 12 months
ElGerges, 2019^34^	Lebanon	Effectiveness of DSME on glycaemic control, self‐efficacy and self‐care activities	Convenience sampling Single site 100 participants (intervention 50: control 50) Mean age 54 50% women	HbA1c (No details on if study was powered) BMI, waist circumference, BP, self‐efficacy, self management, health behaviours	Therapeutic patient education Face to face, group format Delivered by multi‐disciplinary team 1 session, totalling 6 h DSME completion measured at 100% attendance, 50/50 intervention participants finished trial	Therapeutic DSME is efficient in contributing to better glycaemic control, improved self‐efficacy and enhanced self care skills.
Eroglu, 2021^26^	Turkey	Effectiveness of DSME on self‐management and self‐efficacy	Purposive sampling Single site 80 participants (intervention 40; control 40) Mean age 57 37% female	Primary outcome not stated (80% power to detect 0.60 change at *p* = 0.05) Self management skills, self‐efficacy, HbA1c, lipid levels, BMI, fasting blood sugars	Programme name not stated Face to face, 1:1 format Delivered by investigator (background not stated) 1 session and totalling 45 min DSME completion measured at 100% attendance. 40/40 intervention participants finished trial	Self management and self‐efficacy can be increased with DSME Close monitoring of individuals via DSME and telephone follow‐up is effective in achieving metabolic targets.
Ha, 2014^31^	China	Effectiveness of DSME on glycaemic control, self‐efficacy, knowledge about osteoporosis and physical activity	Convenience sampling Single site 50 participants (intervention 25; control 25) Mean age 69 60% female	Primary outcome not stated (80% power to detect medium effect at *p* = 0.05) Knowledge of osteoporosis, diabetes self management, self‐efficacy, dietary calcium intake, level of physical activity, glycaemic control	Name of programme not stated (self‐efficacy based on diabetes osteoporosis management) Face to face, group format Delivered by healthcare professionals 6 sessions totalling 6 h Measure of DSME completion not stated. 23/25 intervention participants finished trial	Study demonstrates the effectiveness of self‐efficacy‐based education in type 2 diabetes
Habibzadeh, 2017^35^	Iran	Effectiveness of DSME on self‐care behaviours	Purposive sampling Single site 90 participants (intervention 45; control 45) Mean age 53 72% female	Self care (80% power to detect medium effect at *p* = 0.05)	Name of programme not stated (based on extended theory of reasoned action) Face to face, group format Delivered by healthcare professionals 8 sessions totalling 12 h Measure of DSME completion not stated. 45/45 intervention participants finished trial	Self management in type 2 diabetes is increased with group DSME which includes discussions and instructive feedback
Jeihooni, 2020^36^	Iran	Effectiveness of DSME on self‐care behaviours in women with type 2 diabetes	Purposive sampling Single site 100 participants (intervention 50; control 50) Mean age not stated 100% female	Primary outcome not stated (not stated) Attitudes, abstract norms, self‐efficacy, behavioural intention, HbA1c.	Name of programme not stated (based on extended theory of reasoned action) Face to face, group format Delivery included a doctor, background of others not stated 10 sessions totalling 10 h Measure of DSME completion not stated. 50/50 intervention participants finished trial	DSME using extended theory of reasoned action significantly improves self‐care behaviours
Khunti, 2012^8^	UK	Benefits of DSME for newly diagnosed type 2 diabetes at 3‐year follow‐up	Purposive sampling Multi‐site including 207 general practices 604 participants (intervention 332; control 272) *subset of those in original trial Mean age 58 46% female	HbA1c (90% power to detect 1% change HbA1c at *p* = 0.05 12 months later) Blood pressure, weight, blood lipid levels, smoking status, physical activity, quality of life, beliefs about illness, depression and emotional impact of diabetes	DESMOND Face to face, group format Delivered by healthcare professionals 1 session totalling 6 h DSME completion measured at 100% attendance. 332 intervention participants followed up at 3 years	A single DSME programme did not demonstrate any sustained biomedical or lifestyle outcome improvements at 3 years Sustained improvements were reported for some illness beliefs.
Maslakpak, 2017^40^	Iran	Effectiveness of family oriented DSME on self‐care, fasting blood sugar, HbA1C and cholesterol	Purposive sampling Single site 90 participants (face to face intervention 30; telephone intervention 30; control 30) Mean age 50 43% female	Self management (80% power at *p* = 0.05) Fasting blood sugar, HbA1c, lipid levels and triglyceride.	Empowerment based diabetes education Face to face and telephone, group format Delivery included a doctor, background of others not stated 10 sessions totalling 10 h Measure of DSME completion not stated. 50/50 intervention participants finished trial	Family orientated DSME has a beneficial effect on self‐care and patient outcomes
McEwen, 2017^27^	USA	Effect on self‐management, self‐efficacy and diabetes distress of a family‐based DSME	Purposive sampling Single site 157 participants (intervention 83; control 74) Mean age 54 65% female	HbA1c (86% power at p0.05 for to detect 1% change) Diabetes knowledge, interpersonal distress	Name of programme not stated Face to face, group and follow‐up 1:1 format Delivered by healthcare professionals 6 sessions totalling 19 h Measure of DSME completion not stated. 43/83 intervention participants finished trial	Culturally relevant DSME interventions can improve diabetes self management and substantiate the need for intensive longer tailored interventions to improve glycaemic control DSME content must account for social norms which can influence diabetes self management
Mohammadi, 2018^37^	Iran	Impact of self‐efficacy DSME based on the health belief model in type 2 diabetes	Random sampling Single site 240 participants (intervention 120; control 120) Mean age 51% female not stated	HbA1c (not stated) Fasting blood glucose, lipid profiles, nutrition knowledge, health beliefs	Name of programme not stated (based on health belief model) Face to face, not stated if 1:1 or group format Delivered by healthcare professionals 8 sessions, totalling 16 h Measure of DSME completion not stated. 100/120 intervention participants finished trial	Tailored DSME can promote self‐care and self‐efficacy in type 2 diabetes DSME should be based on identifying and changing behaviour patterns to reduce associated complications and mortality
Moriyama, 2009^14^	Japan	Effectiveness of a 12‐month DSME intervention on self‐efficacy	Convenience sampling Multi‐site, including two hospitals 75 participants (intervention 50; control 25) Mean age 65 54% female	HbA1c (not stated) Fasting blood glucose, body weight, blood pressure, waist circumference, lipid levels, triglyceride, self‐efficacy, psychological preparatory state, degree of goal attainment, quality of life	Name of programme not stated (based on disease management methodologies) Face to face, 1:1 format Delivered by educator, background not stated 12 sessions, totalling 6 h Measure of DSME completion 100%. 42/50 intervention participants finished trial	DSME successfully improves patient behaviours, goal attainment and self‐efficacy outcomes Future studies should consider telephone and internet alternatives to improve retention
Okafor, 2023^24^	Nigeria	Effectiveness of a DSME programme on QOL for people with type 2 diabetes	Purposive sampling Multi‐site, including 4 hospitals 410 participants (intervention 207; control 203) Mean age 57 57% female	QOL (not stated)	Name of programme not stated (based on disease management methodologies) Face to face, group format with additional phone calls Facilitator background not stated 9 sessions, totalling duration not stated Measure of DSME completion not stated. 198/207 intervention participants finished trial	DSME effective in improving health‐related quality of life and should be recommended in all care plans for type 2 diabetes
Singh, 2022^15^	New Delhi	Effectiveness of group DSME on glycaemic control and self‐care activities	Convenience sampling Single site 158 participants (intervention 77; control 81) Mean age 57 62% female	HbA1c (difference of 0.5% = 80% β = 0.21 response rate based on other studies) Self‐care activities	DSME Face to face, group format Delivered by healthcare professionals 6 sessions, totalling 6 h Measure of DSME completion 100%. 69/77 intervention participants finished trial	Group DSME effectively increases self‐care skills and improves glycaemic control Culturally tailored programmes are better accepted.
Sriklo, 2023^16^	Thailand	Effectiveness of Enhanced Health Literacy through Transformative Learning Program on self‐management and HbA1C in uncontrolled diabetes	Purposive sampling Multi‐site, including 2 hospitals 64 participants (intervention 32; control 32) Mean age 58 63% female	HbA1c (80% power of 0.4 at *p* = 0.05) Self management skills	Transformative learning program Face to face, group format Delivered by nurses 4 sessions, totalling 4 h Measure of DSME completion 100%. 32/32 intervention participants finished trial	Transformative learning should be embedded into existing DSME programmes to support adults with uncontrolled type 2 diabetes reflect on consequences of their condition and recognise need for personal change
Trouilloud, 2013^28^	France	Effectiveness of therapeutic DSME in type 2 diabetes on perceived competence, self‐management behaviours and glycaemic control	Purposive sampling Single site 120 participants (split not clear at baseline) Mean age 57 49% female	Primary outcome measure not stated (Not stated) Self management behaviours, HbA1c	Programme name not stated (therapeutic education) Face to face and self‐directed workbook, 1:1 format with three phone calls Delivery team included diabetologist, dietician, nurse and physical activity counsellor 8 sessions totalling 24 h Measure of DSME completion not stated. Not stated how many from intervention group finished trial. Outliers and non‐attenders at 3 months removed.	A 3‐day therapeutic DSME programme improved glycaemic control, physical activity levels and dietary intake There was a positive impact on perceived competence which is found to be involved in long‐term adherence to self management behaviours
Vincent, 2007^29^	USA	Feasibility and effects of culturally tailored DSME on Mexican Americans with type 2 diabetes	Convenience sampling Single site 20 participants (intervention 10; control 10) Mean age 56 71% female	Not powered Diabetes knowledge, self‐efficacy, blood glucose, HbA1c, weight, BMI	Programme name not stated (followed content from national diabetes education program and ADA guidelines) Face to face, group format Not stated who delivered DSME 8 sessions, totalling 16 h Measure of DSME completion not stated. 9/10 intervention participants finished trial	A culturally tailored DSME program may result in improved outcomes for Mexican Americans with type 2 diabetes.
Wattana, 2007^17^	Thailand	Effectiveness of DSME on HbA1C, coronary heart disease risk and quality of life	Purposive sampling Multi‐site, including two hospitals 157 participants (intervention 114; control 131) Mean age 57 76% female	HbA1c (90% power at *p* = 0.01) Coronary heart disease risk and quality of life	Diabetes self management program Face to face, group with additional 1:1 follow‐up Delivered by researcher, nursing background 3 sessions, total duration not stated Measure of DSME completion 100%. 71/75 intervention participants finished trial	DSME should be included in nurse training as their role is key to promoting uptake Small group education increases self‐efficacy and ability to perform self management behaviours
Wichit, 2017^18^	Thailand	Effectiveness of family orientated DSME on self‐efficacy, glycaemic control and QOL in type 2 diabetes.	Convenience sampling Single site 140 participants (intervention 70; control 70) Mean age 59 (only included patients aged 35 years or older) 73% female	Self management skills (90% power to detect 0.58 change at *p* = 0.05) Self‐efficacy, diabetes knowledge, HbA1c and quality of life	Family orientated self management intervention program Face to face, group format Delivered by nurse 3 sessions, totalling 6 h Measure of DSME completion based on 100%. 67/70 intervention participants finished trial	Family orientated DSME can be translated to other rural communities and has the potential to reduce demands on diabetes educators and health services.
Wong, 2016^30^	Hong Kong	Examine the effect of PEP on QOL, patient enablement and participants global perception of change in their overall health condition	Purposive sampling Multi‐site, 715 participants (intervention 390; control 325) Mean age 64 59% female	Health‐related quality of life (no details of study powered) Patient enablement, global health conditions	Patient empowerment programme (PEP) Face to face, group format Delivered by nurse and community experts 8 sessions totalling 15 h Measure of DSME completion not stated. 226/390 intervention participants finished trial	Patient‐reported outcomes should be integrated with clinical outcomes when evaluating the benefits of DSME

*Note*: DSME completion, attendance and attrition are reported where available. Reporting varied across studies and completion was inferred from participant flow diagrams when not explicitly defined.

**TABLE 2:** Patient‐reported outcome measures.

Author & year	PROM outcome		
	Self management skills	Quality of life	Diabetes‐related distress
Bukhsh, 2022^23^	Baseline self management scores: intervention group 5.92 ± 1.43; control group 5.36 ± 2.24 6 months post DSME: intervention group 6.64 ± 1.58; control group 5.95 ± 1.39 (*p* < 0.01) DSME resulted in a significant improvement in self management scores after 6 months DSMQ		
Cai, 2016^39^	Baseline self management scores: intervention group 19.29 ± 4.35; control group 19.61 ± 4.54 3 months post DSME: intervention group 28.16 ± 1.65; control group 21.38 ± 4.17 (*p* < 0.001) DSME led to a large, consistent and statistically significant improvement in self management scores at 3 months, compared to minimal change in the control group SDSCA—10 item	Baseline physical health‐related quality of life scores: intervention group 46.43 ± 5.56; control group 47.78 ± 6.57 3 months post DSME: intervention group 49.51 ± 5.79, control group 48.09 ± 6.22 (*p* < 0.001) Baseline mental health‐related quality of life: intervention group 42.07 ± 4.59; control group 44.77 ± 6.08 3 months post DSME: intervention group 46.57 ± 6.92; control group 44.38 ± 5.83 (*p* < 0.001) DSME led to statistically significant improvements in both physical and mental health‐related quality of life SF36	
Celik, 2022^33^	Overall self management score for intervention group 86.51 ± 15.34 vs. control 72.34 ± 14.60 (*p* < 0.01) Frequency of education correlates to self‐care skills (*p* = <0.01) Attended one session 82.42 ± 15.60 Attended two sessions 88.89 ± 14.51 Three or more sessions 90.90 ± 14.39 DSME resulted in higher overall self management scores compared to the control group, indicating a meaningful benefit from the DSME program. A significant positive correlation was observed between the frequency of DSME sessions and self‐care skill scores, demonstrating a dose–response relationship between education exposure and self management proficiency DSCS		
Changsieng, 2023^25^	Self management scale made up of three components diet consumption change: 4 weeks intervention group 4.4 (2.8, 6.0) vs. control group 0.4 (−1.0, 1.8) *p* < 0.001, 12 weeks intervention group 5.9 (3.9, 7.8) vs. control group 0.7 (−0.9, 2.7) *p* < 0.001 physical activity change: 4 weeks intervention group 1.1 (0.7, 1.4) vs. control group −0.2 (−0.6, 0.3) *p* < 0.001, 12 weeks intervention group 1.2 (0.9, 1.5) vs. control group −0.5 (−0.8, −0.1) *p* < 0.001 Medication adherence change: 4 weeks intervention group 0.8 (0.1, 1.5) vs. control group 0.1 (−0.6, 0.8) *p* = 0.09, 12 weeks intervention group 1.2 (0.5, 1.8) vs. control group 0.1 (−0.8, 1.0) *p* > 0.05 Significant improvements in both dietary habits and physical activity at 4 and 12 weeks for those completing DSME. Improvements in medication adherence, while higher in the intervention group, did not reach statistical significance Researchers own questionnaire		
Chen, 2019^32^		Baseline quality of life scores: intervention group 0.86; control group 0.89 12 months post DSME: intervention group 0.90; control group 0.93 (*p* = 0.9) At 12 months, quality of life improved slightly in both groups, with no significant difference between them EQ‐5D‐3L	
Davies, 2008^9^		Paper reports no significant differences between groups in any of the 6 dimensions of quality of life at 12 month follow‐up. Analyses result unavailable from www.leicesterdiabetes.org.uk WHO‐QOL‐BREF	Median diabetes distress scores at 8 months post DSME: intervention group 14.1 (4.7–28.1); control group 14.1 (4.7–26.6) Model summary coefficient − 0.05 (−2.73 to 2.63), *p* = 0.97 Median diabetes distress scores at 12 months post DSME: intervention group 14.1 (6.3–28.1); control group 12.5 (4.7–28.1) Model summary coefficient 0.19 (−3.20 to 3.59), *p* = 0.91 Intervention group has a slightly higher non‐significant median distress score than the control group. No evidence that DSME had an impact on reducing diabetes distress at 12 months post DSME PAID
ElGerges, 2019^34^	Baseline self management score: intervention group 2.89 ± 0.78; control group 2.67 ± 0.89 3 months post DSME: intervention group 4.56 ± 0.61; control group 2.48 ± 0.77 (*p* < 0.01) Self management scores improved significantly in the intervention group 3 months post DSME SDSCA—revised 11 item		
Eroglu, 2021^26^	Baseline self management score: Intervention group 3.21 ± 1.63, control group 3.97 ± 1.92 6 Months post DSME: intervention group 8.64 ± 1.39; control group 4.02 ± 1.66 (*p* = 0.001) Self management scores increased significantly in the intervention group 6 months post DSME DSMQ		
Ha, 2014^31^	Baseline self management score: Intervention group 38.9 ± 11.2; control group 40.1 ± 9.7 3 months post DSME: intervention group 49.3 ± 9.3; control group 39.9 ± 9.7 (*p* < 0.001) Self management scores improved significantly at 3 months post DSME SDSCA—revised 11 item		
Habibzadeh, 2017^35^	Baseline self management score: Intervention group 108.8 ± 23.8; control group 103.3 ± 28.8 3 months post DSME: intervention group 149.7 ± 13.1; control group 103.8 ± 24.1 (*p* < 0.001) Self management scores improved significantly 3 months post DSME DSMQ		
Jeihooni, 2020^36^	Baseline self management score: Intervention group 24.5 ± 26.14; control group 58.5 ± 27.19 3 months post DSME: intervention group 12.5 ± 63.39; control group 59.5 ± 29.20 (*p* < 0.001) Intervention groups seem to have less improvement in self management scores than the control group. The large variability within the intervention group makes it difficult to conclude that the intervention had a uniform positive effect across all participants SDSCA—revised 11 item		
Khunti, 2012^8^		Paper reports no significant difference at 3‐year follow‐up. Supplementary file link not working WHO‐QOL‐BREF	Median diabetes distress scores at 3 years post DSME: intervention group 10.0 (3.8–20.0); control 8.8 (2.5–21.3) Model summary coefficient − 0.69 (−3,45 to 2.07), *p* = 0.63 Intervention group has a slightly higher non‐significant median distress score than the control group. No evidence that DSME had an impact on reducing diabetes distress at 3 years post DSME PAID
Maslakpak, 2017^40^	Baseline self management scores: intervention group 49.36 ± 15.74; control group 58.83 ± 18.95 3 months post DSME: intervention group 100.82 ± 14.56; control group 49.46 ± 16.35 (*p* < 0.001) Self management scores increased significantly in the intervention group 3 months post DSME SDSCA—revised 11 item		
McEwen, 2017^27^	Baseline self management scores: intervention group 3.78 (1.4); control group 3.943 (1.4) 6 months post DSME: intervention group 4.63 (0.9); control group 4.36 (1.3) (*p* < 0.001) Self management scores improved significantly in the intervention group 6 months post DSME SDSCA—revised 11 item		Baseline diabetes distress scores: intervention group 15.50 (8.5); control group 12.28 (7.3) 6 months post DSME: intervention group 9.94 (7.0); control group 10.17 (6.6) (*p* < 0.001) Diabetes distress decreased significantly over 6 months post DSME, with the intervention group showing a greater reduction than the control group PAID
Mohammadi, 2018^37^		Baseline quality of life scores: intervention group 52.8 ± 3.42; control group 52.4 ± 3.10 3 months post DSME: intervention group 56.4 ± 3.55; control group 52.8 ± 3.45 (*p* < 0.001) 9 months post DSME: intervention group 57.6 ± 3.99; control group 53.2 ± 3.52 (*p* < 0.001) Quality of life scores improved significantly 3 months post DSME with sustained benefits at 9 months DQOL	
Moriyama, 2009^14^		Baseline quality of life scores: intervention group 3.07 ± 0.06; control group 2.8 ± 0.5 6 months post DSME: intervention group 3.13; control group 2.83 9 months post DSME: intervention group 3.34; control group 2.80 12 months post DSME: intervention group 3.33; control group 2.90 (*p* < 0.04) Quality of life scores improved significantly in the intervention group compared with the control group over 12 months post DSME WHO‐QOL26	
Okafor, 2023^24^		Baseline quality of life scores: intervention groups 52.06 ± 15.02; control groups 56.51 ± 16.44 (significant difference at baseline 0.006) 6 months post DSME: intervention group 64.72 ± 10.96; control group 58.85 ± 15.23 (*p* < 0.001) Although intervention and control groups differed significantly at baseline, quality of life improved substantially more in the intervention group at 6 months post DSME SF‐36	
Singh, 2022^15^	Difference in self management skill scores at 6 months General diet: intervention group 1.72 vs. control group 1.04 (*p* < 0.001) Specific diet: intervention group 1.72 vs. control group 1.14 (*p* < 0.001) Exercise: 2.88 vs. 1.8 (*p* < 0.001) Blood sugar testing: 0.53 vs. control group 0.05 (*p* < 0.01) Foot care: 1.07 vs. 0.15 (*p* < 0.001) Carbohydrate spacing: 1,57 vs. 0.7 (*p* < 0.01) Medication adherence: 0.98 vs. 0.64 (*p* = 0.9) Self management skills significantly improved 6 months post DSME across all self‐care domains except for medication adherence which did not differ between groups SDSCA—revised 11 item		
Sriklo, 2023^16^	Baseline self management score: intervention group 50.69 ± 1.82; control group 50.91 ± 1.94 2 months post DSME: intervention group 57.25 ± 2.31; control group 51.61 ± 1.43 (*p* < 0.001) 3 months post DSME: intervention group 59.56 ± 1.92; control group 51.66 ± 1.97 (*p* < 0.001) Self management scores improved significantly 2 months post DSME and 3 months post DSME The self management behaviours scale		
Trouilloud, 2013^28^	Difference in self management skill scores at 3 months Diet behaviours: intervention group increased from 3.22 to 5.54 vs. control group 2.74 to 3.86 (*p* < 0.01) Physical activity: intervention group increased from 1.77 to 2.29 vs. control group 1.71 to 2.70 (*p* < 0.05) Medication adherence: intervention group increased from 6.40 to 6.39 vs. control group 6.53 to 6.69 (*p* < 0.61) Six months post DSME, intervention group showed significant improvements to dietary recommendations and physical activity levels while the control group remained largely unchanged. Medication adherence was high in both groups at baseline and did not change significantly, consistent with a ceiling effect SDSCA—eight items from revised 11 item tool		
Vincent, 2007^29^	Baseline self management score: intervention group 3.37 (1.0); control group 3.53 (1.0) 4 weeks post DSME: intervention group 4.97 (0.7); control group 4.53 (1.3) (*p* = 0.61) Self management scores increased in both groups 4 weeks post DSME, but the difference did not reach statistical significance SDSCA—revised 11 item		
Wattana, 2007^17^		Baseline quality of life scores: intervention group 60.61 ± 15.27; control 62.38 ± 15.22 6 months post DSME: intervention group 70.43 ± 14.70; control group 61.71 ± 14.73 (*p* < 0.001) DSME led to significant improvements to quality of life scores at 6 months SF‐36	
Wichit, 2017^18^	Baseline self management score: intervention group 80.9 (15.9); control 80.5 (13.4) 5 weeks post DSME: intervention group 96.5 (12.7); control group 80.2 (14.7) (*p* < 0.001) 13 weeks post DSME: intervention group 102.8 (12.1); control group 80.4 (18.1) (*p* < 0.001) DSME led to a sustained and statistically significant improvement in self management skills at 13 weeks post‐intervention SDSCA—revised 11 item	Baseline mental health component score: intervention group 54.1 (8.6); control group 54.3 (7.8) 13 weeks post DSME: intervention group 58.4 (7.2); control group 54.7 (6.5) (*p* < 0.001) Baseline physical health component score: intervention group 46.7 (6.6); control group 48.2 (5.6) 13 weeks post DSME: intervention group 49.9 (6.9); control group 49.4 (5.6) (*p* = 0.2) DSME intervention significantly improved participants' mental health but did not significantly affect physical health scores at 13 weeks post‐intervention SF‐12	
Wong, 2016^30^		*Baseline mental health component* score: intervention group 56.02 ± 9.25; control group 55.93 ± 10.79 1 year post DSME: intervention group 56.46 ± 8.22; control group 54.99 ± 11.06 2‐year post DSME: intervention group 52.57 ± 9.69; control group 52.26 ± 10.93 *Baseline physical health component* score: intervention group 48.93 ± 8.64; control group 43.96 ± 12.11 1‐year post DSME: intervention group 48.72 ± 7.68; control group 44.74 ± 12.14 2‐year post DSME: intervention group 46.97 ± 9.31; control group 43.36 ± 11.33 No significant differences at 12 or 24 months to mental health or physical health‐related quality of life. Results suggest worsening of HRQOL in both groups at 24 months SF‐12	

### 2.6 Critical appraisal

The mixed methods appraisal tool (MMAT) was adopted to assess the methodological quality of included papers allowing for a unified appraisal to our scoping review. As per recommendations, two reviewers (GL and KH) independently judged paper quality against the MMAT screening questions before commencing the tailored questions for randomised control trials (RCTs), non‐randomised studies or quantitative descriptive studies (Table 3).

**TABLE 3:** Mixed methods appraisal table (MMAT).

	Screening questions		Randomised controlled trials					Non‐randomised studies					Quantitative descriptive studies				
	Are there clear research questions?	Do the collected data allow to address the research questions?	Is randomisation appropriately performed?	Are the groups comparable at baseline?	Are there complete outcome data?	Are outcome assessors blinded to the intervention provided?	Did the participants adhere to the assigned intervention?	Are the participants representative of the target population?	Are measurements appropriate regarding both the outcome and intervention (or exposure)?	Are there complete outcome data?	Are the confounders accounted for in the design and analysis?	During the study period, is the intervention administered (or exposure occurred) as intended?	Is the sampling strategy relevant to address the research question?	Is the sample representative of the target population?	Are the measurements appropriate?	Is the risk of nonresponse bias low?	Is the statistical analysis appropriate to answer the research question?
Bukhsh, 2022^23^	Y	Y	Y	CT	Y	N	N										
Cai, 2016^39^	Y	Y						N	Y	Y	Y	Y					
Celik, 2016^33^	CT	N											CT	Y	CT	CT	CT
Changseing, 2023^25^	Y	Y	CT	Y	Y	Y	N										
Chen, 2019^32^	Y	Y											CT	N	Y	N	Y
Davies, 2008^9^	Y	Y						Y	Y	Y	Y	Y					
ElGerges, 2019^34^	Y	Y						N	Y	Y	CT	Y					
Eroglu, 2021^26^	Y	Y	Y	Y	Y	N	Y										
Ha, 2014^31^	Y	Y						N	Y	N	Y	Y					
Habibzadeh, 2017^35^	Y	Y	Y	Y	Y	CT	N										
Jeihooni, 2020^36^	Y	Y	CT	CT	N	CT	Y										
Khunti, 2012^8^	Y	Y						Y	Y	N	Y	Y					
Maslakpak, 2017^40^	Y	Y	Y	Y	Y	Y	Y										
McEwen, 2017^27^	Y	Y	CT	CT	Y	CT	N										
Mohammadi, 2018^37^	Y	Y	CT	N	Y	CT	N										
Moriyama, 2009^14^	N	CT	N	Y	Y	N	N										
Okafor, 2023^24^	N	CT						CT	CT	Y	N	Y					
Singh, 2022^15^	Y	Y	Y	N	Y	N	N										
Sriklo, 2023^16^	Y	Y	Y	Y	Y	N	Y										
Trouilloud, 2013^28^	Y	Y	CT	CT	N	CT	N										
Vincent, 2007^29^	Y	Y						N	Y	N	Y	Y					
Wattana, 2007^17^	Y	Y	CT	Y	Y	N	N										
Wichit, 2017^18^	Y	Y	Y	Y	Y	N	Y										
Wong, 2016^30^	Y	Y						Y	Y	N	Y	Y					

*Note*: MMAT rating: Y, yes ‐ shaded green; N, no ‐ shaded red; CT, cannot tell ‐ shaded yellow.

## 3 RESULTS

A systematic search of three databases identified 1307 records once duplicates were removed, with an additional 12 retrieved through hand‐searching key journals, reference lists and diabetes‐related websites. After full text review of 203 publications, 24 met the final inclusion criteria (Figure 1).

**FIGURE 1:**
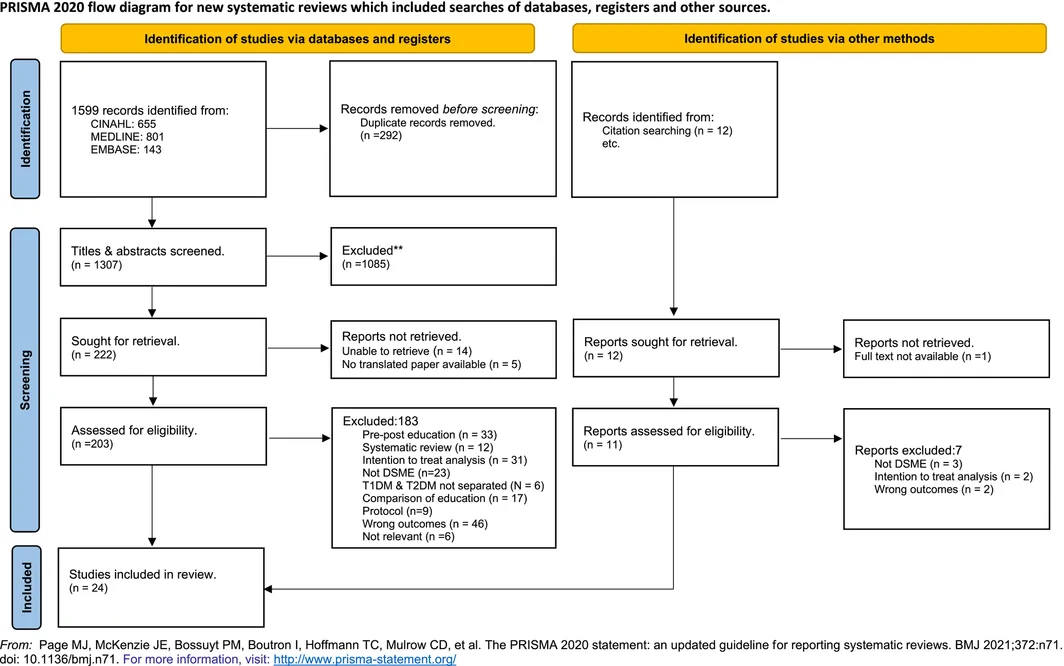
PRISMA flow diagram.

### 3.1 Study characteristics and quality

Many of the studies originated from upper‐middle income countries, predominantly in Asia (*n* = 19). Two RCTs from Pakistan^23^ and India,^15^ and one non‐randomised study from Nigeria,^24^ represented the only contributions from lower‐middle‐income countries. Overall, the review included 14 RCTs, 8 non‐randomised studies (including one 3‐year follow‐up) and 2 quantitative descriptive studies. Among these, 8 RCTs,^14^, ^17^, ^23^, ^25^, ^26^, ^27^, ^28^, ^29^ 4 non‐randomised studies^8^, ^9^, ^30^, ^31^ and 1 quantitative descriptive study reported receiving research funding,^32^ while 6 did not comment on funding.

Publications ranged from 2007^17^ to 2023^16^ peaking in 2017 with a plateau in the last 7 years. All studies were peer‐reviewed and primarily utilised purposive sampling (*n* = 14) or convenience sampling (*n* = 9). Participants were equally distributed between men and women. The mean participant age across studies was 48 years, with sample sizes varying from a small feasibility study (*n* = 20) to large multi‐site descriptive studies (*n* > 1400). Inclusion criteria varied across studies, including factors such as time since diagnosis,^8^, ^9^, ^17^, ^26^, ^27^, ^31^, ^33^, ^34^, ^35^, ^36^ age thresholds,^16^, ^18^, ^31^, ^36^, ^37^ HbA1c 53 mmol/mol (>7%)^16^, ^18^, ^23^, ^25^, ^27^, ^38^, ^39^ and no DSME within the last 12 months.^26^, ^27^, ^29^

### 3.2 DSME programme characteristics

DSME was predominantly delivered in face‐to‐face group sessions with some studies offering one‐to‐one or hybrid alternatives; none included remote or digital options. Whilst there is no standardisation for delivery of DSME, programmes ranged from single sessions (45 min–6 h) to multi‐session programmes totalling up to 24 h making it difficult to conclude if single or multiple sessions programmes are more favourable for supporting changes to PROMs. Education was predominantly delivered by healthcare professionals—most commonly nurses—with occasional delivery by pharmacists or researchers. Several programmes employed behavioural theories (e.g. self‐efficacy, health belief model, extended theory of reasoned action) and tailored content culturally or for family involvement to support engagement and adherence.

Measures of DSME completion were poorly reported and limited by heterogeneity across studies. Ten studies explicitly defined completion as 100% attendance^8^, ^9^, ^14^, ^15^, ^16^, ^17^, ^18^, ^26^, ^34^ while a further two reported no participant attrition, allowing confirmation that all participants completed DSME.^36^, ^40^ In the remaining 12 studies, no clear definition of completion was provided; reviewers inferred likely 100% attendance based on participant flow diagrams or descriptions of intervention receipt. However, across studies, full DSME attendance did not necessarily correspond with trial completion, as several studies reported incomplete follow‐up data despite assumed or reported full intervention participation.

### 3.3 Heterogeneity and methodological quality

Considerable heterogeneity was observed within DSME programme design (session number and length), delivery format (group, one‐to‐one, hybrid) and outcome measurement choice. Inconsistent use of terms such as self care versus self management, beliefs versus attitudes and efficacy versus confidence hindered direct comparisons and interpretation. This variability restricted comparison of results.

Methodological quality was moderate overall but marked by frequent limitations which affected the overall rigor and confidence in findings. Out of 24 included papers, 23 passed the initial screening by clearly defining research questions and collecting appropriate data to address those questions reflecting generally sound study framing. One study^33^ did not explicitly state a research question, resulting in a ‘No’ response to the first screening question and a corresponding ‘Cannot tell’ for the adequacy of collected data, raising concerns about reporting transparency. Many studies listed HbA1c as their primary outcome measure (*n* = 11), with nine being appropriately powered. Five studies did not state primary endpoints,^26^, ^28^, ^31^, ^32^, ^36^ and eight provided no details regarding power calculations^7^, ^14^, ^24^, ^28^, ^32^, ^34^, ^36^, ^37^ thereby limiting the overall methodological quality and ability to detect meaningful changes.

Among the 14 RCTs, only half appropriately performed randomisation, and just five reported participant adherence to assigned interventions and follow‐up data collection.^16^, ^18^, ^26^, ^36^, ^40^ Blinding of outcome assessors was rare, reported in only two studies,^25^, ^40^ increasing the risk of bias. Additionally, two RCTs had groups that were not comparable at baseline,^15^, ^37^ and 4^23^, ^27^, ^28^, ^36^ did not provide enough details to assess this clearly, potentially confounding results. Although most RCTs (86%) reported complete outcome data, these methodological shortcomings limit the validity and reliability of their conclusions.

Only three non‐randomised studies included samples representative of the target populations,^7^, ^8^, ^9^ limiting generalisability. Four studies lacked complete outcome data, introducing potential bias and reducing validity of findings,^7^, ^8^, ^29^, ^35^ one did not provide enough detail to assess, resulting in a ‘Cannot tell’^24^ and two did not account for confounders.^24^, ^34^ Nonetheless, all non‐randomised studies demonstrated high intervention fidelity, ensuring DSME and follow‐up appointments were administered as intended, supporting confidence in their findings.

Both quantitative descriptive studies exhibited poor methodological rigor, with neither providing sufficient detail to assess sampling strategies. Only one stated primary endpoints,^32^ the other provided insufficient detail hindering evaluation and diminished the paper's contribution to the evidence base with a ‘Cannot tell’ rating for four out of five appraisal questions.^33^

### 3.4 Self management skills

Self management skills were the most frequently measured PROM (*n* = 16). Most studies chose validated tools, including the revised Summary of Diabetes Self‐Care Activities (SDSCA) scale (*n* = 10), Diabetes Self‐Management Questionnaire (DSMQ) (*n* = 3), Diabetes Self‐Care Scale (DSCS) (*n* = 1). Of the remaining studies, one adopted a generic self management behaviours scale,^16^ whilst another used a researcher developed tool assessing change in diet consumption, physical activity and medication adherence.^25^ Significant improvements to self management skills were noted in 14 studies at either 3‐month^16^, ^18^, ^31^, ^34^, ^35^, ^39^, ^40^ or 6‐month follow‐up.^23^, ^26^, ^27^ Three studies found improvements were made in some self management domains, with medication adherence showing limited or no change at either 3^25^ or 6 months.^15^, ^28^ Sustained benefits were noted at 6 months in two studies: (1) a pharmacist‐delivered DSME lasting 45 min, (2) a 12‐session programme lasting 24 h delivered by health care professionals. This suggests both formats can be effective in supporting self management skills. A dose–response effect was suggested in one study, whereby higher self management scores correlated with frequency of sessions; however, the number of sessions (1–3) was based on individual clinical need and follow‐up duration was not reported, complicating cross‐study comparisons.^33^ Not all results were positive; one RCT found lower improvements in intervention groups although groups were not matched at baseline^36^ and a pilot study showed improvements at 4 week's post‐intervention; however, these did not reach significance, suggesting longer follow‐up may be required to capture meaningful behavioural changes.^29^ Of the 16 studies assessing self management skills, none reported outcomes for partial‐completers. All studies implied full DSME completion, despite 14 involving multi‐session programmes and 10 reporting incomplete follow‐up data.

### 3.5 Quality of life

Ten studies assessed HRQOL using validated tools such as SF12,^7^, ^18^ SF36,^17^, ^24^, ^39^ EQ‐5D‐3L,^32^ WHO‐QOL‐BREF,^8^, ^9^ WHO‐QOL26,^14^ DQOL.^37^ Significant short‐term improvements were noted at 3 months post‐intervention, particularly in mental health domains,^18^, ^39^ with some sustained improvements at 6^17^, ^24^ and 9 months^37^ post DSME. However, longer‐term outcomes were inconsistent: one study reported significantly improved QOL at 12 months (*p* = 0.04),^14^ whereas another reported minor non‐significant improvements at 12 months.^32^ A large RCT reported no between‐group differences across HRQOL domains at 12 months with similar outcomes in their 3‐year follow‐up.^8^ Similarly, an Asian cohort study failed to report long‐term differences, with a decline in scores from baseline^7^ at 2‐year follow‐up. Of the 10 studies assessing HRQOL, none reported outcomes for partial‐completers. Full intervention completion was implied in seven multi‐session programmes, despite incomplete follow‐up data being reported in these studies.^7^, ^14^, ^17^, ^18^, ^24^, ^32^, ^37^

### 3.6 Diabetes distress

Diabetes distress was assessed in two RCTs, both using the Problem Areas in Diabetes (PAID) scale. A large UK trial (*n* = 824) found no significant effect at 12 months,^9^ a finding supported by its 3‐year follow‐up.^8^ Whereas a smaller USA study (*n* = 157) reported significant reductions 6 months post‐intervention (*p* < 0.001).^27^ No studies reported partial completion data for diabetes distress outcomes. One study involved a single‐session intervention^9^ where partial completion was unlikely, while another reported full programme completion despite substantial attrition at final data collection.^27^

## 4 DISCUSSION

Structured DSME is internationally recommended and considered a cornerstone to supported self management in type 2 diabetes.^5^, ^6^ Existing literature suggests that ‘completing’ DSME often enhances self management skills, improves HRQOL and reduces diabetes distress. However, many studies rely on ITT analyses, diluting results by including participants who did not fully complete the programme. Robust evidence regarding the effectiveness of partially completing DSME remains uncertain, despite internationally adopted benchmarks recommending ≥60% engagement for DSME and prevention programmes. These thresholds are defined by implementation guidance and commissioning requirements rather than evidence of an effective educational dose.^11^, ^12^ This review sought to address this gap by mapping the available literature on the relationship between full or partial completion of DSME and PROMs.

Our review observed short‐term (3 to 6 month) improvements in self management skills where participants completed 100% of the programme. Improvements in self care behaviours, such as dietary changes and glucose monitoring, were more consistently reported than medication adherence, which showed variable responses. This may reflect the complex factors influencing medication‐taking behaviour beyond education alone, such as healthcare access, financial barriers, and social and cultural beliefs.^41^

Short‐term quality of life gains were observed in participants fully completing DSME, although primarily in mental health‐related components, aligning with DSME's role in empowering patients and reducing psychological burden. Nonetheless, the evidence on physical health‐related quality of life was less consistent, potentially due to the relatively short follow‐up periods, potential psychosocial barriers to physical activity or evidence suggesting 37% of patients living with type 2 diabetes are unable to be physically active or exercise normally.^1^ The challenge to draw meaningful conclusions due to high heterogeneity has been highlighted in previous systematic reviews.^42^

Diabetes distress represents the psychological and emotional response caused by the relentless and challenging demands of daily diabetes self management,^43^ thought to affect 20% of people with diabetes in the UK.^1^ Despite this, diabetes distress was evaluated in only two trials, yielding conflicting results. The DESMOND programme, a large UK‐based trial, found no significant changes, while a smaller US study reported reductions at 6 months. Both focused only on participants fully completing DSME, providing limited and contradictory evidence and underscoring a critical gap in the literature, especially given the recognised importance of addressing diabetes distress for improved self management and clinical outcomes.^44^

A key finding of this review is the absence of robust evidence on the effects of partial DSME completion. Despite benchmarks recommending minimum engagement thresholds (e.g. 10% attendance, 60% completion),^11^, ^12^ no studies clearly reported outcomes for participants partially completing DSME (1–99%). This scoping review included studies with DSME programmes ranging from single‐session interventions to multi‐session programmes delivered over 12 months. Single‐session DSME programmes were included and recorded as 100% completion unless stated otherwise; however, we acknowledge that the ability to report on partial completion within such designs is limited. This reflects inherent design limitations in many DSME trials, where outcome assessments occur at fixed time points and discontinuation from the educational programme is closely linked with loss to follow‐up. As a result, partial completion is often analytically indistinguishable from non‐completion, restricting the ability to examine dose–response relationships and limiting guidance on minimum effective engagement levels.

A strength of this review is the focus on per‐protocol analyses, allowing clearer isolation of DSME completion effects compared to ITT analysis which combines completers and partial‐completers. Additionally, the inclusion of diverse study designs and geographical regions, including middle‐income countries, enriches the evidence base. Nonetheless, several limitations warrant caution in interpreting these findings. While RCTs remain central to evaluating the overall effectiveness of DSME, alternative designs such as implementation studies, service evaluations and observational cohorts offer advantages for describing real‐world patterns of programme attendance and engagement. However, when PROMs are the primary outcomes of interest, these designs are limited by substantial missing data, as participants who partially complete DSME are often least likely to complete PROM assessments. Accordingly, trials specifically designed to examine dose–response relationships, with strategies to capture PROMs across varying levels of attendance, may be better suited to addressing questions about the benefits, or lack thereof, of partial DSME completion.

Behavioural change programmes like DSME have been described as complex interventions where it is often difficult to define the active ingredient(s) which make one programme more or less successful than the next.^10^ A lack of standardisation in DSME and considerable heterogeneity in programme design, delivery formats, duration and educator backgrounds complicate direct comparison and synthesis. Furthermore, the effectiveness of DSME may be influenced by the education provider.^45^ Despite the lack of representation in this review, there is a growing role for digital programmes like Healthy Living Type 2 Diabetes^46^ and MyDESMOND.^47^ Study design precluded them from being included within this review however both noted a similar lack of long‐term improvements to PROMs.^46^, ^47^

The studies included within this review exhibited notable weaknesses in methodology—such as incomplete reporting, inadequate randomisation, limited blinding and poor representativeness. As a result, the overall quality of the evidence was poor to moderate. To improve the reliability and applicability of future research, studies should ensure thorough and transparent reporting of methods and outcomes, prioritise representative sampling and implement strategies to reduce bias. These improvements will enhance both the comparability of DSME programmes and strengthen their validity.

Based on the findings of this review, future DSME trials should routinely report detailed attendance data and predefine categories of programme exposure, including partial completion. Alongside standard intention‐to‐treat analyses, pre‐specified secondary analyses comparing outcomes across levels of attendance would enable examination of potential dose–response relationships. These analyses would complement, rather than replace, ITT approaches by clarifying how varying engagement relates to patient‐reported outcomes.

One key recommendation is for authors to adhere to established guidelines like the Standards for Quality Improvement Reporting Excellence in Education (SQUIRE‐EDU)^48^ when submitting educational research. Adopting these guidelines, specifically the checklist's emphasis on detailed description of the educational intervention, measurement of exposure and fidelity, and use as a pre‐requisite for journals would improve the clarity, completeness and transparency in DSME studies, facilitating interpretation of results and reducing redundancy across studies.

Moreover, inconsistent use of outcome measures and variable reporting of DSME attendance and completion further hindered the ability to compare findings across studies. Several studies exhibited methodological flaws such as inadequate randomisation, small sample sizes and incomplete reporting of outcomes, which limit the ability to draw robust conclusions from the existing literature.

Additionally, the predominance of studies from upper‐middle‐income countries raises concerns about the generalisability of findings to low‐ and middle‐income settings where the burden of diabetes is rapidly increasing. More research is needed in these countries to better understand the effectiveness of DSME completion in diverse contexts.

Overall, this scoping review identifies key gaps in the evidence base, particularly the lack of robust data suggesting partial completion of DSME can provide a benefit to patients' self management skills, HRQOL or diabetes distress. While these findings currently limit the ability to define evidence‐based minimum engagement thresholds, they highlight important deficiencies in study design, reporting and evaluation that must be addressed before such guidance can be developed. In line with NICE guidance,^5^ greater standardisation should relate to core components and reporting frameworks rather than prescriptive delivery, allowing DSME to remain responsive to individual and group needs. As the first review to collate evidence across these outcomes, it provides a foundation for informing future research priorities and has clear implications for policy and practice. Specifically, the findings have implications for implementation and service level guidance, including the Centers for Disease Control and Prevention National standards for DSMES,^49^ and the Health Innovation Network toolkits for optimal DSME delivery.^50^ By highlighting the lack of consistent reporting of programme attendance, completion rates and participant engagement, this review can inform the refinement of national and international guidelines aimed at optimising DSME implementation, evaluation and equitable access.

## FUNDING INFORMATION

No funding was received for this work.

## CONFLICT OF INTEREST STATEMENT

GI is the National NIHR Research Delivery Network lead for General Practice. JW reports consultancy / advisory board work for the pharmaceutical industry contracted via the University of Liverpool in the last 36 months (no personal payment) for Alnylam, Amgen, AstraZeneca, Boehringer Ingelheim, Cytoki, Kailera, Lilly, Menarini, Metsera, Napp, Novo Nordisk, Pfizer, Prosciento, Response Pharmaceuticals, Rhythm Pharmaceuticals, Saniona, Shionogi and Ysopia; funding for clinical trials from Amgen, AstraZeneca and Novo Nordisk and personal honoraria / lecture fees from AstraZeneca, Boehringer Ingelheim, Medscape, Novo Nordisk and Menarini. JW is past president of the World Obesity Federation, a member of the Association for the Study of Obesity, Diabetes UK, EASD, ADA, Society for Endocrinology and the Rank Prize Funds Nutrition Committee. From 2009 to 2024 he was national lead for the Metabolic and Endocrine Speciality Group of the UK NIHR Clinical Research Network. GL, DH and KH report no conflicts of interest.

## Supporting information


**Data S1.** CINAHL PLUS search strategy.

**Data S2.** MEDLINE search strategy.
