# Supplementary material for: Real‐world completion of diabetes self management education and its impact on patient‐reported outcomes: A scoping review
Source: Diabet Med. 2026 Apr 3;43(10):e70312. doi: 10.1111/dme.70312 (PMC13643630; doi:10.1111/dme.70312)
Supplement: Supplementary file 1 [file DME-43-e70312-s001.docx]

MEDLINE search strategy

| [**Search ID#**](javascript:__doPostBack('ctl00$ctl00$MainContentArea$MainContentArea$historyControl$ReorderHistoryLink','')) | | **Search Terms** |
| --- | --- | --- |
|  | S24 | S11 AND S23 |
|  | S23 | S12 OR S13 OR S14 OR S15 OR S16 OR S17 OR S18 OR S19 OR S20 OR S21 OR S22 |
|  | S22 | health related quality of life or hrqol or quality of life or qol |
|  | S21 | (MH "Self Care") |
|  | S20 | self-care activities or self care or daily activities |
|  | S19 | diabetes distress or diabetes related distress or diabetes-specific emotional distress |
|  | S18 | patient reported outcome measures or prom or patient reported outcomes |
|  | S17 | (MH "Patient-Reported Outcomes") |
|  | S16 | social determinants of health |
|  | S15 | indicies of multiple deprivation |
|  | S14 | health deprivation |
|  | S13 | (MH "Health Inequities") OR (MH "Social Determinants of Health") |
|  | S12 | (MH "Social Deprivation") |
|  | S11 | S1 OR S10 |
|  | S10 | S4 AND S9 |
|  | S9 | S5 OR S6 OR S7 OR S8 |
|  | S8 | structured education |
|  | S7 | self management education or education or diabetes self management education program |
|  | S6 | patient education or patient self management education or diabetes education or diabetes training or diabetes knowledge |
|  | S5 | (MH "Diabetes Education") OR (MH "Patient Education") OR (MH "Patient Education as Topic") OR (MH "Teach-Back Communication") OR (MH "Education") OR (MH "Teaching") |
|  | S4 | S2 OR S3 |
|  | S3 | diabetes mellitus type 2 or diabetes type 2 or t2dm or type 2 diabetic or t2d or diabetes 2¨ or niddm or non-insulin dependent diabetes mellitus |
|  | S2 | (MH "Diabetes Mellitus, Type 2") |
|  | S1 | (MH "Diabetes Mellitus, Type 2/ED") |
