# Supplementary material for: Real‐world completion of diabetes self management education and its impact on patient‐reported outcomes: A scoping review
Source: Diabet Med. 2026 Apr 3;43(10):e70312. doi: 10.1111/dme.70312 (PMC13643630; doi:10.1111/dme.70312)
Supplement: Supplementary file 2 [file DME-43-e70312-s002.docx]

# CINAHL PLUS search strategy

| [**Search ID#**](javascript:__doPostBack('ctl00$ctl00$MainContentArea$MainContentArea$historyControl$ReorderHistoryLink','')) | | **Search Terms** | |
| --- | --- | --- | --- |
| S25 | S11 AND S24 | |  |
| S24 | S12 OR S13 OR S14 OR S15 OR S16 OR S17 OR S18 OR S19 OR S20 OR S21 OR S22 OR S23 | |  |
| S23 | health related quality of life or hrqol or quality of life or qol | |  |
| S22 | (MH "Self Care") | |  |
| S21 | self-care activities or self care or daily activities | |  |
| S20 | diabetes distress or diabetes related distress or diabetes-specific emotional distress | |  |
| S19 | patient reported outcome measures or prom or patient reported outcomes | |  |
| S18 | (MH "Patient-Reported Outcomes") | |  |
| S17 | social determinants of health | |  |
| S16 | indicies of multiple deprivation | |  |
| S15 | indicies of multiple deprivation | |  |
| S14 | health deprivation | |  |
| S13 | (MH "Health Inequities") OR (MH "Social Determinants of Health") | |  |
| S12 | (MH "Social Deprivation") | |  |
| S11 | S1 OR S10 | |  |
| S10 | S4 AND S9 | |  |
| S9 | S5 OR S6 OR S7 OR S8 | |  |
| S8 | structured education | |  |
| S7 | self management education or education or diabetes self management education program | |  |
| S6 | patient education | |  |
| S5 | (MH "Diabetes Education") OR (MH "Patient Education") | |  |
| S4 | S2 OR S3 | |  |
| S3 | diabetes mellitus type 2 or diabetes type 2 or t2dm or type 2 diabetic or t2d or diabetes 2¨ | |  |
| S2 | (MH "Diabetes Mellitus, Type 2") | |  |
| S1 | (MH "Diabetes Mellitus, Type 2/ED") | |  |
